# Multi-modal molecular and spatial profiling reveals NNT as a prognostic biomarker in obesity-associated colorectal cancer

**DOI:** 10.1007/s00535-025-02339-4

**Published:** 2025-12-28

**Authors:** Sungjin Park, Jae-Ghi Lee, Ilkyu Park, Soyeon Jeong, Jungsuk An, Jisup Kim, Myunghee Kang, Seungyoon Nam, Jung Ho Kim

**Affiliations:** 1 Department of Genome Medicine and Science, Gachon Institute of Genome Medicine and Science, College of Medicine, Gachon University Gil Medical Center, Gachon University, Incheon, 21565 Republic of Korea; 2 Gachon Medical Research Institute, Gachon Biomedical Convergence Institute, Gachon University Gil Medical Center, Gachon University College of Medicine, Incheon, 21565 Republic of Korea; 3 Department of Pathology, Ewha Womans University Mokdong Hospital, Ewha Womans University College of Medicine, Seoul, 07985 Republic of Korea; 4 Department of Pathology, Gil Medical Center, Gachon University College of Medicine, Gachon University, Incheon, 21565 Republic of Korea; 5 Department of Pathology, Dongkang Medical Center, Ulsan, 44455 Republic of Korea; 6 Department of Health Sciences and Technology, Gachon Advanced Institute for Health Sciences and Technology (GAIHST), Gachon University, Incheon, 21999 Republic of Korea; 7 Department of Internal Medicine, Gachon University Gil Medical Center, College of Medicine, Gachon University, Incheon, 21565 Republic of Korea; 8 Department of Translational-Clinical Medicine, Gachon Advanced Institute for Health Sciences and Technology (GAIHST), Gachon University, Incheon, 21999 Republic of Korea

**Keywords:** Obesity-associated colorectal cancer, NNT, Biomarker, Survival prediction, TNM classification

## Abstract

**Background:**

Obesity is a known risk factor for colorectal cancer (CRC), but its impact on prognosis and tumor biology remains unclear. This study aimed to identify molecular biomarkers that reflect obesity-associated tumor characteristics and stratify patient outcomes.

**Methods:**

We conducted a multi-step analysis integrating transcriptomic data, clinical validation, and spatial profiling. Candidate genes were first screened in the TCGA-COADREAD dataset based on expression trends across normal, healthy-weight CRC, and obese CRC samples. Prognostically relevant genes were then validated in an independent cohort using immunohistochemistry (IHC). Finally, spatial transcriptomic analysis using GeoMx DSP was performed to elucidate the tumor microenvironment associated with the top candidate.

**Results:**

Among six shortlisted genes, NNT showed a significant association with overall survival specifically in obese patients and was validated at the protein level by IHC. High NNT expression was independent of TNM stage and associated with improved prognosis. Spatial transcriptomic profiling revealed that NNT-high tumors were enriched for antioxidant, apoptotic, and metabolic programs, while oncogenic and proliferative pathways were suppressed. These patterns suggest that NNT contributes to a redox-balanced and metabolically adaptive tumor state.

**Conclusions:**

Through integrative molecular and spatial analyses, NNT was identified as a potential prognostic biomarker in obesity-associated CRC. This study highlights the importance of combining clinical data with spatial transcriptomics to uncover context-specific tumor biology.

**Supplementary Information:**

The online version contains supplementary material available at 10.1007/s00535-025-02339-4.

## Introduction

Colorectal cancer (CRC) is the most common malignancy of the gastrointestinal tract and the second leading cause of cancer-related mortality worldwide, with approximately 1.9 million new cases and over 935,000 deaths reported in 2020 [1]. Despite advances in treatment and early detection, prognostic assessment in CRC still relies heavily on anatomical staging systems such as TNM. These systems, while clinically useful, do not capture the biological heterogeneity of tumors or host-specific factors such as metabolic status, which may profoundly affect patient outcomes [2].

Obesity has emerged as a significant global health issue and a well-established risk factor for CRC [3, 4]. Paradoxically, however, several studies have reported improved survival outcomes in obese cancer patients compared to those with normal weight—a phenomenon known as the “obesity paradox” [5, 6]. The biological basis of this paradox remains poorly understood. One hypothesis is that obesity may define a distinct molecular or microenvironmental subtype of cancer, rather than conferring a uniform protective effect. However, the lack of obesity-specific cell lines and animal models has hindered efforts to mechanistically dissect this relationship, highlighting the need for human tissue-based molecular investigations.

A promising avenue lies in the identification of biomarkers that not only stratify prognosis but also reflect obesity-associated tumor biology [7]. In this context, mitochondrial redox regulation has gained attention, as metabolic imbalance and oxidative stress are hallmarks of both obesity and cancer [8]. Obesity-driven mitochondrial dysfunction can elevate reactive oxygen species (ROS), which promote tumor progression by damaging DNA and activating oncogenic signaling pathways, such as MAPK and NF-κB [9, 10]. Although cells maintain redox homeostasis through antioxidant systems, persistent oxidative stress may foster tumor adaptation and immune evasion [11, 12]. Thus, redox regulation represents a critical link between obesity and cancer biology and may offer insights into novel prognostic biomarkers.

In this study, we aimed to identify molecular biomarkers specifically associated with obesity-related CRC and investigate their clinical and biological relevance. We performed transcriptomic analysis of public CRC datasets stratified by body mass index (BMI) to identify candidate genes linked to obesity status. Findings were validated in an independent CRC patient cohort using immunohistochemistry and survival analysis. Given the central role of overall survival in clinical decision-making, we specifically evaluated the prognostic value of candidate biomarkers. To further explore the tumor-intrinsic and microenvironmental roles of the most promising candidate, we employed spatial transcriptomic profiling using the GeoMx Digital Spatial Profiler [13, 14]. This integrative approach was designed to uncover clinically meaningful biomarkers and elucidate potential mechanisms underlying obesity-associated CRC prognosis.

## Materials and methods

### Study overview

This study was conducted to identify and validate prognostic biomarkers specific to obesity-associated colorectal cancer (CRC) using a three-phase design: biomarker discovery in a public transcriptomic dataset, clinical validation in a patient cohort, and spatial transcriptomic analysis to explore underlying biological mechanisms. The initial discovery phase utilized gene expression data from The Cancer Genome Atlas (TCGA) CRC cohort stratified by body mass index (BMI). The validation phase involved immunohistochemical (IHC) analysis of tumor tissues from an independent cohort of CRC patients. Finally, spatial transcriptomics using the GeoMx Digital Spatial Profiler (DSP) was performed on formalin-fixed paraffin-embedded (FFPE) samples to assess microenvironmental and pathway-level changes associated with the biomarker of interest.

### TCGA CRC cohort and candidate gene discovery

Transcriptomic and clinical data for the TCGA CRC cohort were obtained from the UCSC Cancer Genomics Browser [15–17]. Among 434 CRC cases, 417 samples from patients aged 40 years or older were selected. From these, 414 primary tumor and adjacent normal tissue samples were identified, and 268 samples with available weight and height data at the time of diagnosis were included in the final analysis. These were stratified into three groups based on BMI: adjacent normal tissue from healthy-weight patients (AN), tumor tissue from healthy-weight patients (HT; BMI < 25 kg/m^2^), and tumor tissue from overweight or obese patients (OT; BMI ≥ 25 kg/m^2^) [18].

To identify obesity-associated molecular patterns, genes were screened for expression trends that changed consistently across the three groups. Specifically, we defined upward-trending genes as those with increasing median expression from AN to HT to OT, and downward-trending genes as those with decreasing median expression in that same order. This stepwise trend filtering was applied to prioritize genes potentially linked to obesity-related tumor progression.

### Clinical validation cohort, tissue microarray, and immunohistochemistry

The clinical validation cohort consisted of 456 patients who underwent surgical resection for primary CRC at Gachon University Gil Medical Center (GMC) between April 2010 and January 2013. Patients with recurrent CRC, prior bowel-altering surgeries, preoperative chemotherapy or radiotherapy, or previous cancer diagnoses were excluded. After applying exclusion criteria, 448 patients with preserved tumor blocks were included for analysis. Tissue microarrays (TMAs) were constructed from formalin-fixed, paraffin-embedded (FFPE) tumor samples [19, 20]. Two 2-mm cores were extracted from each tumor and embedded in recipient paraffin blocks.

IHC was performed using the BOND III automated stainer (Leica Biosystems, Newcastle, UK) and the Bond Polymer Refine Detection system (DS9800; Leica Biosystems, Melbourne, Australia). The following primary antibodies were used: anti-NNT (1:250, PA5-52,118, Abcam, Cambridge, UK), anti-PANK3 (1:200, ab247294, Abcam, Cambridge, UK), anti-PPARGC1B (1:500, ab118603, Abcam, Cambridge, UK), anti-RTL6 (1:20, ab31917, Abcam, Cambridge, UK), anti-FAM220A (1:100, ab229254, Abcam, Cambridge, UK), and anti-TMEM9 (1:200, ab236864, Abcam, Cambridge, UK). Staining intensity was scored on a scale from 0 to 3 for most markers. For antibodies that exhibited weak or inconsistent staining patterns across samples, a 0-to-2 scoring scale was used to reflect limited detection sensitivity. For analysis, scores of 0–1 were considered low expression and 2–3 as high expression.

The study was approved by the Institutional Review Board of Gachon University Gil Medical Center (GBIRB2016-318) and conducted in accordance with the Declaration of Helsinki.

### GeoMx DSP data processing and statistical analysis

This analysis included colorectal cancer (CRC) patients from the validation cohort at Gachon University Gil Medical Center. GeoMx Digital Spatial Profiling (DSP) was performed on formalin-fixed paraffin-embedded (FFPE) samples from 89 patients, all of whom were part of the validation cohort. Patients were selected according to the following criteria: (1) availability of paired tumor and adjacent normal tissues with adequate RNA integrity for ROI selection; (2) complete BMI and clinicopathologic information; (3) representative coverage across TNM stages I–IV and BMI categories (healthy weight, 18.5– < 25 kg/m^2^; overweight/obese, ≥ 25 kg/m^2^; excluding underweight cases); and (4) absence of neoadjuvant therapy, preoperative radiotherapy, or severe necrosis affecting tissue quality. Cases that failed NanoString QC or lacked sufficient area for ROI selection were excluded.

For each case, tumor and adjacent normal regions were annotated by an experienced gastrointestinal pathologist based on H&E reference slides and morphology markers. To enable obesity-stratified spatial analysis, we extended the TCGA-based classificaiton by defining adjacent normal tissues from overweight/obese patients as ON. Accordingly, a total of 115 ROIs were analyzed, comprising 16 AN, 24 ON, 33 HT, and 42 OT regions. This sampling strategy ensured balanced representation across BMI and tissue types while minimizing technical bias. ROIs were retained if they met the following quality thresholds: ≥ 1,000 raw reads; ≥ 80% trimmed; ≥ 50% stitched; ≥ 50% aligned reads; ≥ 50% sequencing saturation; negative controls > geometric mean 1; ≥ 3 nuclei; and ≥ 100 µm^2^ area. Probe QC excluded targets whose geometric-mean ratio to all probes was ≤ 0.1 or that failed Grubbs’ test in ≥ 20% of segments, and individual outlier segments were excluded locally. The limit of quantification (LOQ) was defined as the mean negative-probe signal + 2 SD. To correct for ROI-specific variation (e.g., area, mRNA input, hybridization), data were normalized using third-quartile (Q3) normalization. The final dataset comprised 115 ROIs × 9,490 genes. Obese and non-obese cohorts were further stratified by NNT expression levels, selecting the top and bottom 10 samples in each group for differential expression analysis. All downstream analyses, including hierarchical clustering (pheatmap) and gene set enrichment analysis (fgsea), were conducted in R version 4.3.2.

### Survival analysis

Survival analysis was conducted separately for the discovery and validation cohorts. In the TCGA dataset, gene candidates that showed significant expression trends were further evaluated for prognostic relevance. Five-year overall survival (OS) was analyzed based on mRNA expression using Kaplan–Meier curves [21]. In contrast, in the GMC validation cohort, five-year event-free survival (EFS) and disease-specific survival (DSS) were assessed according to biomarker protein expression measured by IHC as only OS data were available in the TCGA dataset. This approach allowed for a more clinically detailed evaluation of patient outcomes. In this study, events were defined as follows: for OS, death from any cause; for EFS, recurrence, metastasis, or death; and for DSS, death specifically attributed to colorectal cancer. Statistical methods for both cohorts included log-rank tests and Cox proportional hazards models. Subgroup analysis was stratified by BMI category (HT vs. OT) and biomarker expression level. Multivariate Cox proportional hazards models were adjusted for age and sex to minimize confounding effects. Age was treated as a continuous variable and sex as a binary variable (male/female). All analyses were performed using SPSS version 25.0.

## Results

### Transcriptomic discovery of prognostic biomarker candidates

**Fig. 1:**
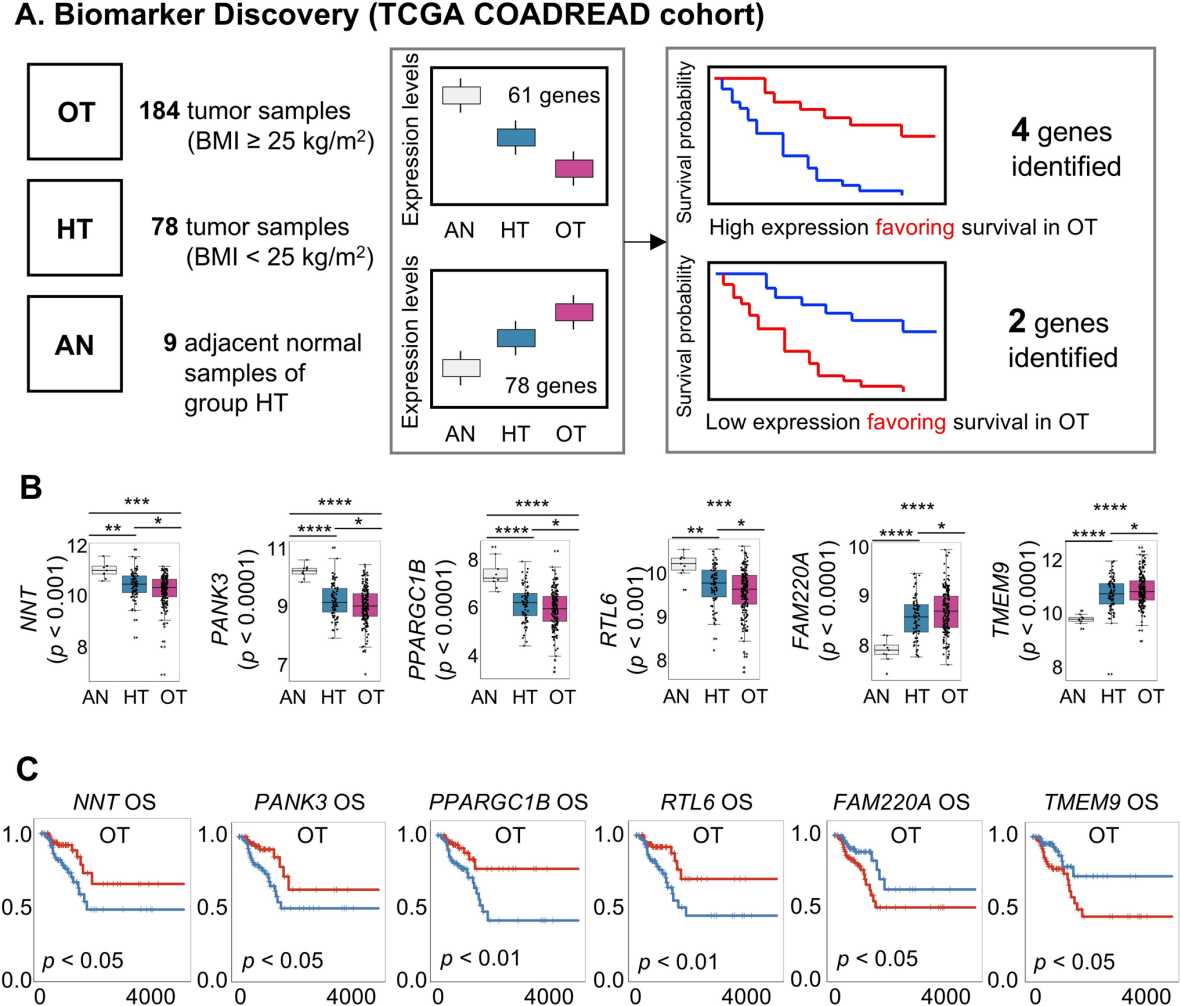
Discovery of obesity-associated prognostic biomarker candidates in colorectal cancer (CRC). **A** Schematic overview of gene expression trend analysis using TCGA COADREAD data. Samples were stratified into adjacent normal (AN), tumor tissue from healthy-weight CRC patients (HT), and tumor tissue from overweight/obese CRC patients (OT). A total of 139 genes showed monotonic expression changes across the AN–HT–OT axis, with 61 genes displaying a downward trend and 78 genes showing an upward trend. **B** Bar plots showing expression patterns of six candidate genes (*NNT*, *PANK3*, *PPARGC1B*, *RTL6*, *FAM220A*, and *TMEM9*) across the three groups. Four genes showed a downward trend (higher in AN, lower in OT), and two genes showed an upward trend (lower in AN, higher in OT). **C** 5-year overall survival analysis in the OT group revealed six genes significantly associated with prognosis. High expression of downward-trend genes (*NNT, PANK3, PPARGC1B, RTL6*) and low expression of upward-trend genes (*FAM220A, TMEM9*) were associated with favorable survival outcomes. OT, tumor tissue samples from overweight and obese patients with CRC (BMI ≥ 25 kg/m^2^); HT, tumor tissue samples from healthy weight patients with CRC (body mass index [BMI] < 25 kg/m^2^); AN, adjacent normal tissue samples from group HT

We performed a comparative transcriptomic analysis using the TCGA COADREAD dataset to identify prognostic biomarkers associated with obesity in colorectal cancer (CRC). Samples were classified into three groups—adjacent normal tissue (AN), tumor tissue from healthy-weight CRC patients (HT), and tumor tissue from overweight/obese CRC patients (OT). A total of 139 genes demonstrated a consistent monotonic trend in expression across the AN–HT–OT axis, with 61 showing a downward pattern and 78 showing an upward pattern (Fig. 1A). Of these, six representative genes—NNT, PANK3, PPARGC1B, RTL6 (downward), and FAM220A, TMEM9 (upward)—were selected based on consistent trends and survival relevance. Their expression profiles showed progressive changes across AN, HT, and OT groups, as visualized in bar plots (Fig. 1B), reflecting obesity-associated transcriptional shifts.

These six candidates also demonstrated statistically significant associations with overall survival in the OT group: NNT, PANK3, PPARGC1B, and RTL6 (downward trend) and FAM220A and TMEM9 (upward trend) (Fig. 1C). In the OT group, higher expression of downward-trend genes and lower expression of upward-trend genes correlated with improved survival outcomes. This pattern was not observed in the HT group, suggesting a potential obesity-specific prognostic effect. Accordingly, these genes were selected for further validation by immunohistochemistry (IHC) in an independent CRC patient cohort.

### Clinical and pathological characteristics by NNT expression

Clinical validation was conducted in an independent cohort of 448 CRC patients from Gachon University Gil Medical Center. Of these, 127 (28.3%) were classified as having obesity-associated CRC (BMI ≥ 25 kg/m^2^), while 321 (71.7%) were healthy-weight CRC patients (Table 1). The mean patient age was 65.1 ± 11.4 years, with 60.0% male and 40.0% female. BMI values differed markedly between the groups, with healthy-weight patients showing a mean BMI of 21.7 ± 2.1 kg/m^2^ and overweight/obese patients showing a mean BMI of 27.3 ± 2.1 kg/m^2^. (t test, p < 0.0001). Most tumors were located in the sigmoid colon or rectum and were moderately differentiated. Based on TNM staging, 15.1% were stage I, 32.6% stage II, 33.0% stage III, and 11.8% stage IV.

**Table 1:** Baseline characteristics

	Total (*n* = 448)
Age	65.1 ± 11.4
Sex	
Male	269 (60.0%)
Female	179 (40.0%)
BMI	
BMI < 25 (kg)	21.7 ± 2.1
BMI ≥ 25 (kg)	27.3 ± 2.1
Laboratory findings	
Hemoglobin (g/dL)	12.2 ± 2.4
WBC (10^3^/μL)	7542 ± 2684.7
CEA (ng/mL)	82.3 ± 408.6
Histology	
Size (mm)	52.1 ± 21.9
Location	
Cecum	10 (2.1%)
Ascending colon	77 (15.9%)
Hepatic flexure	8 (1.6%)
Transverse colon	29 (6.0%)
Splenic flexure	3 (0.6%)
Descending colon	12 (2.5%)
Sigmoid-descending	5 (1.0%)
Sigmoid colon	112 (23.1%)
Rectosigmoid colon	82 (16.9%)
Rectum	110 (22.7%)
Pathology	
WD	27 (5.6%)
MD	383 (79.0%)
PD	18 (3.7%)
Mucinous	18 (3.7%)
SRC	2 (0.4%)
TNM stage	
I	73 (15.1%)
II	158 (32.6%)
III	160 (33.0%)
IV	57 (11.8%)

*BMI* Body mass index, *CEA* carcinoembryonic antigen, *MD* moderately differentiated carcinoma, *PD* poorly differentiated carcinoma, *SRC* signet ring cell carcinoma, *TNM* tumor, node, metastasis, *WBC* white blood cells, *WD* well differentiated carcinoma

To further explore clinical differences between the obesity-associated and healthy-weight CRC groups, we compared clinicopathological characteristics based on BMI classification (Table 2). No significant differences were observed between the two groups with respect to age, sex, diabetes mellitus, family history, anemia, white blood cell counts, serum CEA levels, tumor location, tumor differentiation, or TNM stage. In contrast, the prevalence of smoking was lower in the overweight/obese CRC group than in the healthy-weight group (9.4% vs 17.8%). Both univariate and multivariate analyses indicated an inverse association between smoking and overweight/obese CRC (univariate *p* = 0.028; multivariable OR 0.466, 95% CI 0.229–0.949, *p* = 0.035). This trend is consistent with the well-established inverse relationship between smoking and body weight. [22, 23]. In addition, we assessed the clinicopathological associations of the six candidate genes identified in the TCGA analysis. Univariate and multivariate analyses revealed distinct clinical correlations for several of these genes. Specifically, PANK3 was associated with TNM stage; PPARGC1B with serum CEA levels and tumor differentiation; RTL6 with anemia and tumor differentiation; FAM220A with both tumor differentiation and TNM stage; and TMEM9 with anemia, tumor differentiation, and TNM stage (Online Resources 1–5). NNT expression showed no significant association with conventional clinicopathological variables (BMI < 25 vs ≥ 25: univariate *p* = 0.293; multivariable OR 0.768, 95% CI 0.491–1.202, *p* = 0.249), supporting that its prognostic impact is largely independent of baseline features (Table 3).

**Table 2:** Clinical characteristics of patients with colorectal cancer (CRC)

Variables		Univariate			Multivariate	
	Total (*n* = 448)	Healthy-weight CRC (*n* = 321)	Obese CRC (*n* = 127)	*p* value	OR (CI)	*p* value
Age (yrs)		65.5 ± 11.6	63.9 ± 10.5	0.741		0.915
< 65	196 (43.8%)	142 (44.2%)	54 (42.5%)		1 (Reference)	
≥ 65	252 (56.3%)	179 (55.8%)	73 (57.3%)		1.025 (0.658–1.593)	
Sex				0.261		0.553
Male	269 (60.0%)	198 (61.7%)	71 (55.9%)		1 (Reference)	
Female	179 (40.0%)	123 (38.3%)	56 (44.1%)		1.143 (0.736–1.773)	
Diabetes mellitus				0.515		0.554
No	358 (79.9%)	259 (80.7%)	99 (78.0%)		1 (Reference)	
Yes	90 (20.1%)	62 (19.3%)	28 (22.0%)		1.175 (0.688–2.004)	
Smoking				0.028		0.035
No	379 (84.6%)	264 (82.2%)	115 (90.6%)		1 (Reference)	
Yes	69 (15.4%)	57 (17.8%)	12 (9.4%)		0.466 (0.229–0.949)	
Family history				0.693		0.644
No	389 (86.8%)	280 (87.2%)	109 (85.8%)		1 (Reference)	
Yes	59 (13.2%)	41 (12.8%)	18 (14.2%)		1.156 (0.625 – 2.137)	
Anemia				0.224		0.123
No	230 (51.3%)	159 (49.5%)	71 (55.9%)		1 (Reference)	
Yes	218 (48.7%)	162 (50.5%)	56 (44.1%)		0.703 (0.450–1.100)	
WBC counts				0.650		0.556
Normal	376 (83.9%)	271 (84.4%)	105 (82.7%)		1 (Reference)	
Abnormal	72 (16.1%)	50 (15.6%)	22 (17.3%)		1.190 (0.667 – 2.122)	
Serum CEA				0.997		0.890
Normal	328 (73.2%)	235 (73.2%)	93 (73.2%)		1 (Reference)	
Abnormal	120 (26.8%)	86 (26.8%)	34 (26.8%)		0.966 (0.590–1.581)	
Tumor location				0.422		0.684
LCC	323 (72.1%)	228 (71.0%)	95 (74.8%)		1 (Reference)	
RCC	125 (27.9%)	93 (29.0%)	32 (25.2%)		0.902 (0.479–1.483)	
Tumor differentiation				0.771		0.642
Differentiation	409 (91.3%)	293 (91.3%)	117 (91.5%)		1 (Reference)	
Undifferentiation	38 (8.5%)	28 (33.3%)	10 (7.9%)		0.830 (0.379–1.820)	
TNM stage				0.465		0.537
I/II	231 (51.6%)	169 (52.6%)	62 (48.8%)		1 (Reference)	
III/IV	217 (48.4%)	152 (47.4%)	65 (51.6%)		1.147 (0.742–1.773)

**Table 3:** Clinicopathologic significance of NNT expression

Variables		Univariate			Multivariate	
	Total (*n* = 448)	NNT (*n* = 290), Low	NNT (*n* = 158), High	*p* value	OR (CI)	*p* value
Age (yrs)				0.116		0.067
< 65	196 (43.8%)	119 (41.0%)	77 (48.7%)		1 (Reference)	
≥ 65	252 (56.3%)	171 (59.0%)	81 (51.3%)		0.677 (0.446–1.028)	
Sex				0.562		0.524
Male	269 (60.0%)	177 (61.0%)	92 (58.2%)		1 (Reference)	
Female	179 (40.0%)	113 (39.0%)	66 (41.8%)		1.148 (0.751–1.754)	
Diabetes mellitus				0.577		0.548
No	358 (79.9%)	234 (80.7%)	124 (78.5%)		1 (Reference)	
Yes	90 (20.1%)	56 (19.3%)	34 (21.5%)		1.170 (0.702–1.950)	
Smoking				0.927		0.489
No	379 (84.6%)	245 (84.5%)	134 (84.8%)		1 (Reference)	
Yes	69 (15.4%)	45 (15.5%)	24 (15.2%)		0.809 (0.444–1.282)	
Family history				0.597		0.629
No	389 (86.8%)	250 (86.2%)	139 (88.0%)		1 (Reference)	
Yes	59 (13.2%)	40 (13.8%)	19 (12.0%)		0.862 (0.672–1.575)	
Anemia				0.982		0.963
No	230 (51.3%)	149 (51.4%)	81 (51.3%)		1 (Reference)	
Yes	218 (48.7%)	141 (48.6%)	77 (48.7%)		1.010 (0.662–1.541)	
WBC counts				0.331		0.444
Normal	376 (83.9%)	247 (85.2%)	129 (81.6%)		1 (Reference)	
Abnormal	72 (16.1%)	43 (14.8%)	29 (18.4%)		1.236 (0.718–2.128)	
Serum CEA				0.136		0.100
Normal	328 (73.2%)	219 (75.5%)	101 (69.0%)		1 (Reference)	
Abnormal	120 (26.8%)	71 (24.5%)	49 (31.0%)		1.471 (0.929–2.330)	
Tumor location				0.646		0.826
LCC	323 (72.1%)	207 (71.4%)	116 (73.4%)		1 (Reference)	
RCC	125 (27.9%)	83 (28.6%)	42 (26.6%)		0.949 (0.597–1.511)	
Tumor differentiation				0.119		0.086
Differentiation	409 (91.3%)	259 (89.3%)	150 (94.9%)		1 (Reference)	
Undifferentiation	38 (8.5%)	30 (10.3%)	8 (5.1%)		0.483 (0.210–1.110)	
TNM stage				0.485		0.400
I/II	231 (51.6%)	146 (50.3%)	85 (53.8%)		1 (Reference)	
III/IV	217 (48.4%)	144 (49.7%)	73 (46.2%)		0.837 (0.553–1.267)	
BMI				0.293		0.249
BMI < 25	321 (71.7%)	203 (70.0%)	118 (74.7%)		1 (Reference)	
BMI ≥ 25	127 (28.3%)	87 (30.0%)	40 (25.3%)		0.768 (0.491–1.202)	

### NNT as a potential independent prognostic marker in obesity-related CRC

**Fig. 2:**
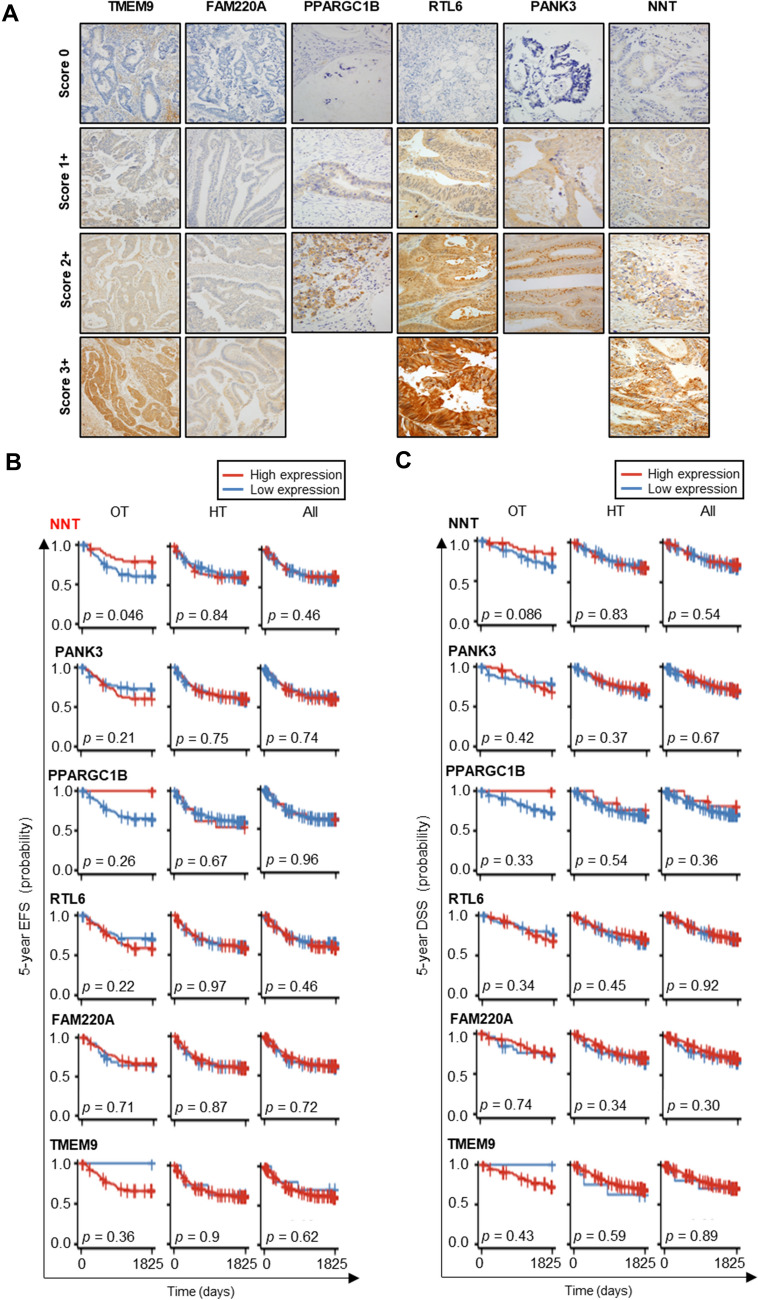
Discovery and clinical validation of new biomarker candidate specific for obesity-associated colorectal cancer. **A** Representative immunohistochemical staining of six candidate proteins—NNT, PANK3, PPARGC1B, RTL6, FAM220A, and TMEM9—in colorectal cancer tissue. Staining intensity and distribution were scored semi-quantitatively as follows: score 0, faint and rare immunostaining; score 1 + , focal and weak staining; score 2 + , moderate and diffuse staining; and score 3 + , strong and diffuse staining. **B**–**C** 5-year event-free survival (**B**) and 5-year disease-specific survival (**C**) according to the expression of six candidates (NNT, PANK3, PPARGC1B, RTL6, FAM220A, and TMEM9) in the group OT, group HT, and all patients with CRC; FAM220A, family with sequence similarity 220 member A; NNT, Nicotinamide nucleotide transhydrogenase; PANK3, pantothenate kinase 3; PPARGC1B, peroxisome proliferator-activated receptor gamma coactivator 1-beta; RTL6, retrotransposon Gag like 6; TMEM9, transmembrane protein 9

To validate our findings, we performed IHC and survival analysis, using the GMC cohort. First, we stratified the 448 patients into OT and HT groups according to BMI (cutoff value of 25 kg/m^2^), and each group was further classified into high and low subgroups based on IHC expression (Fig. 2A). Thereafter, we performed survival analysis to evaluate the association between six protein expression, BMI status, and 5-year event-free survival (EFS) and disease-specific survivals (DSS). Among the six candidate proteins in the GMC cohort, survival analysis validated NNT as the sole protein with a significant association with patient survival. In the OT group, survival analysis revealed that the rate of 5-year EFS was lower in the lower expression subgroup (NNT_Low_) compared to that in the higher expression subgroup (NNT_High_), and the HR of NNT_Low_ group versus the NNT_High_ group was 2.20 in the multivariate model (CPH model adjusted for age and sex, 95% confidence interval [CI]: 1.01–4.80, *p* < 0.05). In the HT group and the entire cohort, the 5-year EFS did not differ between the NNT_Low_ and NNT_High_ subgroups. In addition, the other five candidates showed no difference in the 5-year EFS and DSS (Fig. 2C and D), regardless of expression and BMI status, suggesting that NNT may be a specific prognostic biomarker for overweight CRC patients.

### Survival impact of NNT expression in obesity-associated CRC

**Fig. 3:**
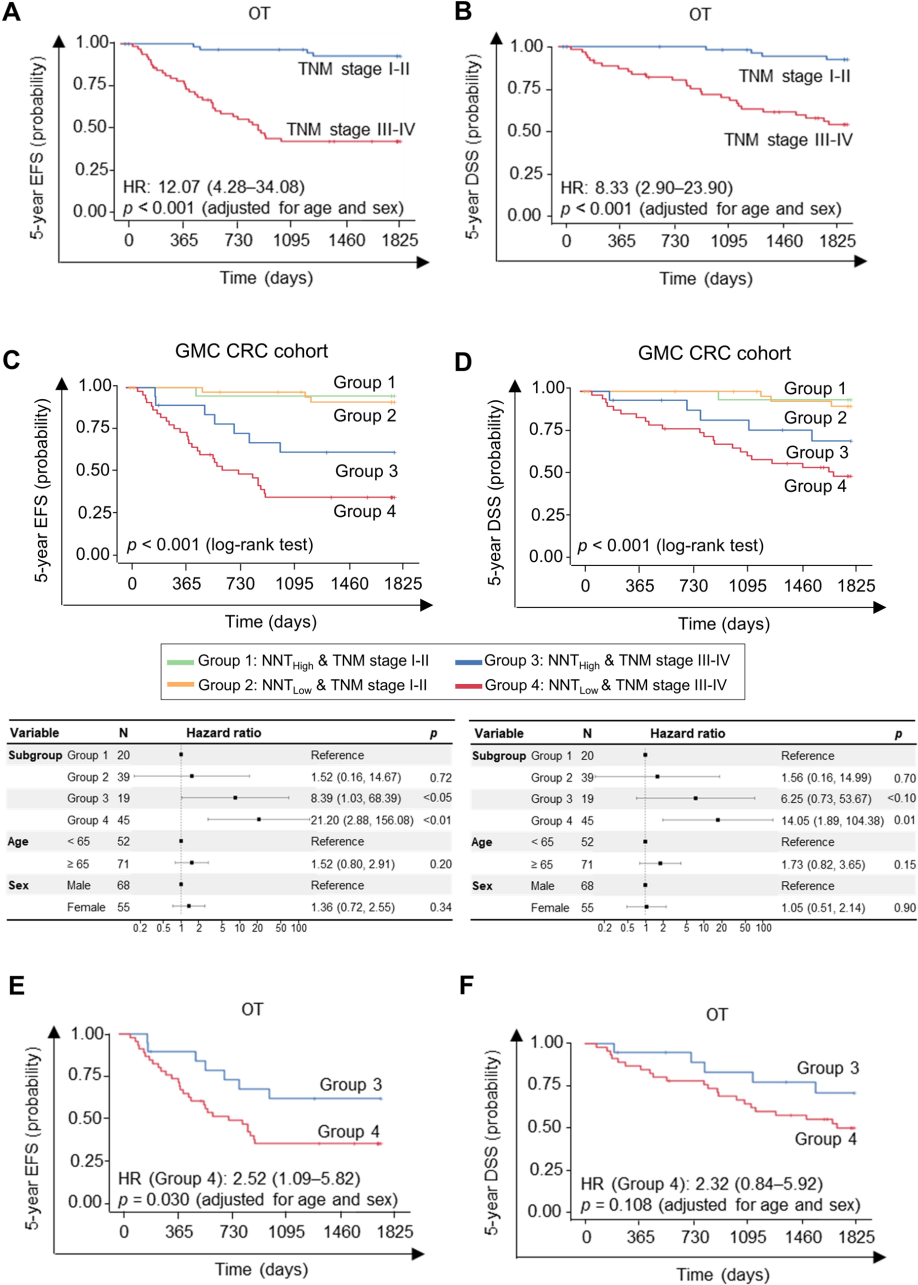
Multivariate test of NNT in the GMC cohort. **A**–**B** 5-year event-free survival (EFS) (**A**) and 5-year disease-specific survival (DSS) (**B**) considering only tumor node metastasis (TNM) stage in the OT group. Survival analysis with TNM stage alone. **C**–**D** GMC cohort multivariate test of 5-year EFS (**C**) and 5-year DSS (**D**) combined with NNT and TNM staging system of OT patients. **E**–**F** 5-year EFS (**E**) and 5-year DSS (**F**) according to NNT expression at the same TNM stage (III-IV). Panels **A**–**B** illustrate the individual prognostic impact of TNM stage alone, while panels **C**–**F** show the combined analysis integrating NNT expression with TNM staging

We first evaluated the prognostic relevance of TNM stage in obesity-associated CRC (OT) patients. As expected, patients with late-stage disease (stage III–IV) had significantly poorer 5-year EFS and disease-specific survival DSS compared to those with early-stage disease (stage I–II). In multivariate analysis, the HR for EFS in stage III–IV versus I–II was 12.07 (95% CI: 4.28–34.08, *p* < 0.001), and the HR for DSS was 8.33 (95% CI: 2.90–23.90, *p* < 0.001) (Fig. 3A and B). Importantly, the individual prognostic effects of NNT expression and TNM stage are presented separately in Figs. 2 and 3 (for NNT) and Figs. 3A–B (for TNM), both demonstrating significant associations with survival.

Given that NNT expression was independent of conventional clinicopathologic factors, we investigated whether combining NNT expression with TNM staging could improve prognostic stratification in obesity-associated CRC. To this end, patients were categorized into four groups: Group 1 (high NNT expression and early-stage disease [Stage I–II]), Group 2 (low NNT expression and early-stage), Group 3 (high NNT expression and advanced-stage [Stage III–IV]), and Group 4 (low NNT expression and advanced-stage). Multivariate survival analysis revealed a clear and stepwise stratification of survival outcomes across these four groups. Group 1 exhibited the most favorable prognosis, followed by Group 2 and Group 3, while Group 4 demonstrated the worst 5-year event-free survival (EFS) and disease-specific survival (DSS). In particular, Group 4 showed a markedly elevated risk of adverse outcomes compared to Group 1, with an EFS hazard ratio (HR) of 21.20 (95% CI: 2.88–156.08, *p* < 0.01) and a DSS HR of 14.05 (95% CI: 1.89–104.38, *p* = 0.01) (Fig. 3C and D). These results demonstrate that integrating NNT expression with TNM stage enables more refined and clinically meaningful risk stratification, allowing for a more accurate prediction of prognosis than either variable alone.

Within the subgroup of patients with advanced-stage disease (TNM stage III–IV), further stratification by NNT expression revealed significant survival differences. Patients with low NNT expression (Group 4) showed markedly lower 5-year event-free survival (EFS) compared to those with high NNT expression (Group 3), with a hazard ratio (HR) of 2.52 (95% CI: 1.09–5.82; *p* = 0.030). A similar trend was observed for disease-specific survival (DSS) (HR: 2.32, 95% CI: 0.84–5.92), though not statistically significant (Fig. 3E and F). These results indicate that NNT expression stratifies prognosis even within the same TNM stage, suggesting that its combination with staging criteria may enhance prognostic resolution in patients with obesity-associated CRC.

### Spatially resolved metabolic and signaling reprogramming associated with NNT expression in obese CRC

**Fig. 4:**
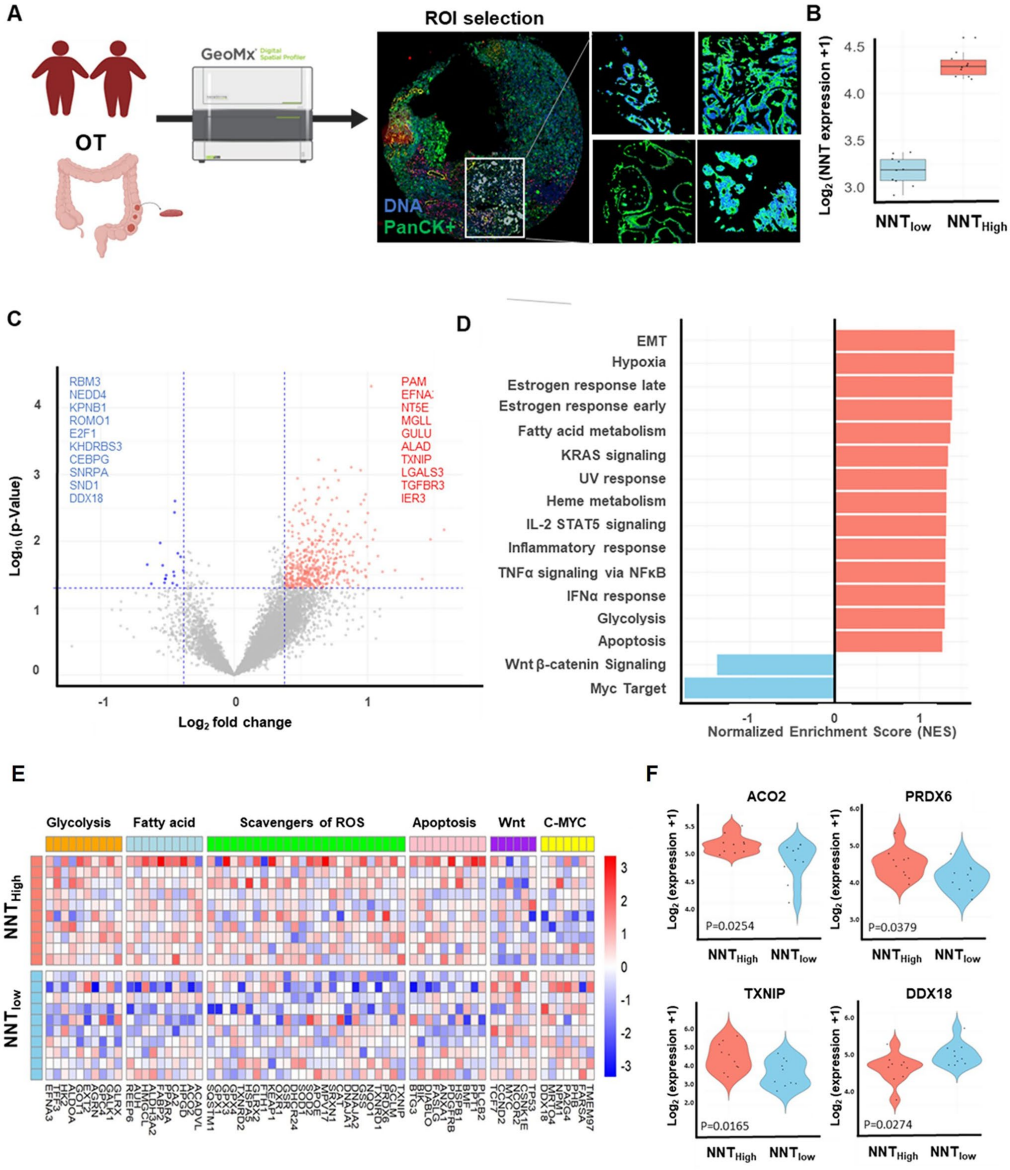
Analysis of NNT expression in obese colorectal cancer (CRC) patients using GeoMx spatial transcriptomics technology. **A** GeoMx analysis of FFPE tissue from obese CRC patients (OT group): Formalin-fixed paraffin-embedded (FFPE) tissue was collected from obese CRC patients, and GeoMx technology was used for spatial transcriptomics. The tumor epithelial regions were isolated based on DNA and PanCK markers to ensure accurate targeting of the tumor cells. **B** Boxplot of NNT expression groups: The patients were divided into high NNT expression and low NNT expression groups. **C** Volcano plot of gene expression differences between NNT high and low groups. **D** GSEA analysis results: The normalized enrichment scores (NES) derived from GSEA analysis are presented. **E** Heatmap of gene expression related to metabolic pathways, such as glycolysis, fatty acid metabolism, scavengers of reactive oxygen species (ROS), apoptosis, Wnt /β-catenin signaling, and c-Myc signaling. **F** Boxplots of *ACO2, PRDX6, TXNIP*, and *DDX18* genes

To elucidate spatial transcriptomic changes associated with NNT, we performed GeoMx DSP on FFPE samples from the validation cohort, profiling both tumor and adjacent normal regions across BMI-defined groups (AN, ON, HT, and OT), and stratifying tumors into NNT_High_ and NNT_Low_ where indicated (Fig. 4A, B; Supplementary Fig. S1). To further delineate obesity-related transcriptional differences, NNT expression was compared across four spatial groups (AN, ON, HT, and OT) representing adjacent normal and tumor tissues stratified by BMI. A significant group-level difference was observed (ANOVA *p* < 0.0001; Supplementary Fig. S1A), showing a progressive decrease from AN → ON → HT → OT. This finding indicates that obesity-related downregulation of NNT emerges already in adjacent normal tissues and becomes more pronounced in tumors. In NNT_High_ tumors (Fig. 4C), transcripts involved in metabolic and apoptotic pathways—including *PAM, EFNA1, NT5E, MGLL, GULO, ALAD, TXNIP, LGALS3, TGFBR3*, and *IER3*—were significantly upregulated, suggesting increased glycolytic activity, enhanced fatty acid metabolism, and heightened apoptotic signaling. In contrast, genes associated with cellular proliferation and poor prognosis—such as *RBM3, NEDD4, KPNB1, ROMO1, E2F1, KHDRBS3, CEBPG, SNRPA, SND1*, and *DDX18*—were markedly downregulated. These transcriptomic shifts indicate that NNT_High_ tumors adopt a metabolically active yet less proliferative tumor phenotype.

Pathway-level analysis revealed selective enrichment of hallmark programs related to epithelial–mesenchymal transition, hypoxia, fatty acid metabolism, glycolysis, heme metabolism, UV response, and apoptosis in NNT_High_ tumors (Fig. 4D). Conversely, oncogenic pathways including Wnt/β-catenin and c-Myc signaling were significantly suppressed. These spatially confined alterations suggest that elevated NNT expression fosters a redox-protective, metabolically reprogrammed microenvironment that may counterbalance tumor-promoting signals. Consistent with this interpretation, spatial mapping of the antioxidant response showed localized upregulation of reactive oxygen species (ROS)-scavenging genes—such as *CAT, GPX1–3, SOD1–2, TXNIP*, and *PRDX6*—within NNT_High_ tumors (Fig. 4E). Notably, *ACO2* (*p* = 0.0254), *PRDX6* (*p* = 0.0379), and *TXNIP* (*p* = 0.0165) were significantly enriched in NNT_High_ tumors, implicating enhanced mitochondrial metabolism and redox buffering capacity. In contrast, *DDX18* (*p* = 0.0274) was predominantly expressed in NNT_Low_ tumors, consistent with elevated proliferative potential (Fig. 4F). Within the HT (non-obese tumor) compartment, we further examined four NNT-associated genes—DDX18, PRDX6, TXNIP, and ACO2—to investigate context-specific redox regulation (Supplementary Fig. S1B). DDX18 was higher in NNT-high cases, PRDX6 showed a mild increase in NNT-high tumors, whereas TXNIP and ACO2 were unchanged. These data suggest that NNT selectively modulates antioxidant and metabolic genes, supporting a context-dependent adaptive redox response rather than uniform antioxidant activation.

Together, these spatially resolved transcriptomic insights reveal that high NNT expression reprograms discrete tumor niches toward enhanced energy metabolism and oxidative stress management, while concurrently dampening oncogenic signaling. These findings provide a mechanistic basis for the observed favorable prognostic impact of NNT in obesity-associated CRC.

## Discussion

Obesity is a well-established risk factor for colorectal cancer (CRC) [24], yet the biological mechanisms linking metabolic status to tumor behavior remain poorly defined. Traditional staging systems such as TNM [25] focus on anatomical spread but fail to account for obesity-associated molecular heterogeneity that may influence prognosis [2]. Moreover, paradoxical observations—such as improved outcomes in some obese cancer patients (the so-called “obesity paradox”) [5, 6]—further highlight the need for biomarkers that reflect the unique tumor biology in this context and support individualized prognostic assessment.

To address this gap, we conducted a multi-platform analysis designed to identify and clinically validate a prognostic biomarker specific to obesity-associated CRC. Using transcriptomic data from the TCGA CRC cohort stratified by BMI, we identified six candidate genes—*TMEM9, PANK3, PPARGC1B, RTL6, FAM220A*, and *NNT*—that exhibited directional expression changes across adjacent normal tissue, healthy-weight tumors, and obesity-associated tumors. Importantly, we linked these trends to patient survival, ensuring that selection was based not only on biological consistency but also clinical relevance. Because only overall survival (OS) data were available in the TCGA cohort, whereas event-free survival (EFS) and disease-specific survival (DSS) data were available in our validation cohort, different endpoints were used according to data availability. This strategy also provided greater clinical granularity for survival assessment. This survival-centered approach, extended through event-free and disease-specific survival validation in a large, independent TMA-based IHC cohort (*n* = 448), reinforces the translational potential of our findings. Although the difference did not reach statistical significance, NNT expression tended to be higher in obese CRC patients, consistent with the transcriptomic pattern observed in TCGA and GeoMx datasets.

Among the six candidates, only NNT demonstrated a significant association with prognosis at the protein level, specifically in obese patients. Furthermore, NNT expression was independent of standard clinicopathologic features such as TNM stage or tumor grade, underscoring its value as an orthogonal biomarker. Previous studies have linked high NNT expression to enhanced mitochondrial redox homeostasis and altered survival outcomes in other cancer types, supporting its broader prognostic relevance beyond CRC [26]. NNT is a mitochondrial enzyme that supports redox homeostasis by generating NADPH, which is essential for detoxifying reactive oxygen species (ROS). This function is particularly relevant in obesity, where oxidative stress is elevated and may influence tumor progression [27]. When integrated with TNM staging, NNT enabled refined prognostic stratification, particularly in advanced-stage (III–IV) patients. This layered classification approach provides a practical framework for identifying high-risk patients who may benefit from intensified surveillance or tailored therapies. Inclusion of ON samples in the spatial analysis revealed that NNT expression is already reduced in adjacent normal tissues of overweight/obese patients, with the lowest levels observed in obese tumors (Supplementary Fig. S1A). Moreover, within the HT compartment, selective changes in DDX18 and PRDX6 (Supplementary Fig. S1B) support the concept that NNT fine-tunes specific elements of the redox network to adapt to metabolic stress rather than driving a global antioxidant response.

To explore the biological significance of NNT, we employed spatial transcriptomic profiling using the GeoMx Digital Spatial Profiler. This platform allowed us to spatially compare gene expression patterns between NNT-high and NNT-low tumors, enabling tumor compartment level analysis not achievable by bulk or single-cell methods. Based on our findings, we propose the following hypothesis: in obese CRC, tumors with high NNT expression establish a redox-stabilized and metabolically regulated state [28], characterized by elevated expression of genes involved in fatty acid metabolism, oxidative stress mitigation, and apoptosis. This observation aligns with previous evidence that NNT supports mitochondrial NADPH production, thereby facilitating ROS detoxification under oxidative or hypoxic stress [29]. However, antioxidant and redox-regulating pathways, including GPX1–3 and PRDX6, are known to exert context-dependent roles in cancer. While maintaining redox balance can protect against excessive oxidative damage, the same mechanism may also promote tumor cell survival under metabolic or inflammatory stress [30–32]. Therefore, the increased expression of antioxidant genes in NNT-high tumors likely reflects an adaptive response to the oxidative environment associated with obesity, rather than a purely tumor-suppressive process. This interpretation is further supported by recent findings showing that NNT sustains mitochondrial NADPH production and redox cycling, enabling metabolic adaptation under stress conditions [33].

In contrast, NNT-low tumors exhibited increased expression of proliferative and oncogenic pathways, such as c-Myc and Wnt/β-catenin signaling. Interestingly, although EMT and hypoxia-related signatures—typically associated with tumor aggressiveness—were also observed in NNT-high tumors, they co-occurred with redox-balancing and pro-apoptotic programs. Taken together, these findings suggest that NNT-mediated redox regulation in obese CRC may act as a double-edged mechanism—mitigating oxidative stress while simultaneously supporting metabolic adaptation—ultimately contributing to a more controlled tumor phenotype.

Our spatial transcriptomics analysis, uniquely enabled by spatial transcriptomics, further demonstrated that antioxidant-related genes (*ACO2* [34], *PRDX6* [35], *TXNIP* [36]) were concentrated in NNT-high tumors, while proliferative and immune-evasive signals (*DDX18* [37]) were confined to NNT-low tumors. Such intratumoral compartmentalization cannot be captured by bulk or single-cell RNA sequencing, highlighting the essential advantage of spatial platforms in decoding tumor heterogeneity and microenvironmental dynamics.

While we acknowledge the absence of functional in vitro or in *vivo* validation as a limitation, our integrative use of large-scale transcriptomic discovery, survival-focused clinical validation, and spatially resolved mechanistic analysis presents a compelling, patient-derived evidence framework. Additionally, the validation cohort was derived from a single institution, which may limit generalizability; thus, external confirmation in multi-center or prospective studies will be important. Nonetheless, this strategy directly connects biomarker biology with oncologic outcomes, the most decisive metric in clinical oncology.

**Fig. 5:**
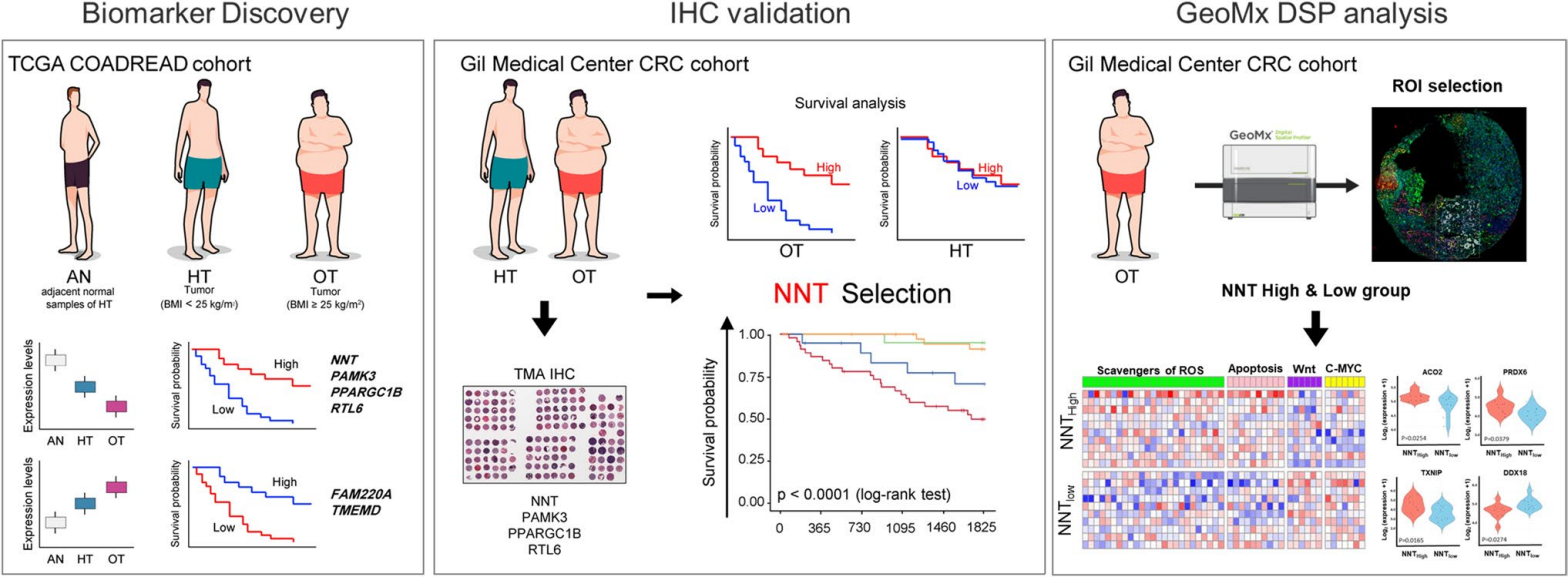
Overview of study workflow and key findings. Biomarker discovery using the TCGA COADREAD cohort identified six genes associated with prognosis in obesity-associated colorectal cancer (CRC). NNT was selected and validated in the Gil Medical Center CRC cohort using tissue microarray immunohistochemistry (IHC) and survival analysis. GeoMx digital spatial profiling of the CRC cohort characterized NNT-high and NNT-low tumors, revealing differences in pathways including scavengers of reactive oxygen species (ROS), apoptosis, Wnt/β-catenin signaling, and c-Myc target expression. CRC, colorectal cancer; IHC, immunohistochemistry; ROS, reactive oxygen species

Taken together, these findings suggest that NNT overexpression in obesity-associated CRC may modulate tumor–immune interactions and antioxidant responses, contributing to favorable prognosis. The overall study workflow and proposed mechanistic model are summarized in Fig. 5. In conclusion, NNT is a novel and clinically validated prognostic biomarker for obesity-associated CRC. Its expression level is strongly associated with survival outcomes—the most clinically meaningful indicator of oncologic efficacy—with low NNT indicating worse prognosis and high NNT correlating with favorable survival. Notably, NNT enabled additional prognostic refinement even within the same TNM stage, supporting its value in personalized risk stratification and treatment planning. Supported by spatial transcriptomic insight and validated through IHC on a large clinical cohort, NNT offers both mechanistic depth and practical feasibility. These strengths highlight its potential for rapid integration into precision oncology strategies tailored for obese CRC patients.

## Supplementary Information

Below is the link to the electronic supplementary material.

Supplementary file1 Fig. S1 (A) NNT expression across AN, ON, HT, and OT (boxplot with ANOVA p-value, ROI numbers). (B) HT compartment gene expression (DDX18, PRDX6, TXNIP, ACO2) by NNT level (t-test p-values) (JPG 259 KB)

Supplementary file2 (XLSX 29 KB)

## References

[1] Sung H, Ferlay J, Siegel RL, et al. Global cancer statistics 2020: GLOBOCAN estimates of incidence and mortality worldwide for 36 cancers in 185 countries. CA Cancer J Clin. 2021;71:209–49. 10.3322/caac.21660. 33538338 10.3322/caac.21660

[2] Hendlisz A, Deleporte A, Delaunoit T, et al. The prognostic significance of metabolic response heterogeneity in metastatic colorectal cancer. PLoS ONE. 2015;10:e0138341. 10.1371/journal.pone.0138341. 26421426 10.1371/journal.pone.0138341 PMC4589397

[3] Heindel JJ, Lustig RH, Howard S, et al. Obesogens: a unifying theory for the global rise in obesity. Int J Obes. 2024;48:449–60. 10.1038/s41366-024-01460-3. 10.1038/s41366-024-01460-3 PMC10978495 38212644

[4] Lin X, Li H. Obesity: epidemiology, pathophysiology, and therapeutics. Front Endocrinol (Lausanne). 2021;12:706978. 10.3389/fendo.2021.706978. 34552557 10.3389/fendo.2021.706978 PMC8450866

[5] Lennon H, Sperrin M, Badrick E, et al. The obesity paradox in cancer: a review. Curr Oncol Rep. 2016;18:56. 10.1007/s11912-016-0539-4. 27475805 10.1007/s11912-016-0539-4 PMC4967417

[6] Li Y, Li C, Wu G, et al. The obesity paradox in patients with colorectal cancer: a systematic review and meta-analysis. Nutr Rev. 2022;80:1755–68. 10.1093/nutrit/nuac005. 35182150 10.1093/nutrit/nuac005

[7] Abdulla A, Sadida HQ, Jerobin J, et al. Unraveling molecular interconnections and identifying potential therapeutic targets of significance in obesity-cancer link. J Natl Cancer Cent. 2025;5:8–27. 10.1016/j.jncc.2024.11.001. 40040878 10.1016/j.jncc.2024.11.001 PMC11873641

[8] Black HS. Oxidative stress and ROS link diabetes and cancer. J Mol Pathol. 2024;5:96–119.

[9] Reuter S, Gupta SC, Chaturvedi MM, et al. Oxidative stress, inflammation, and cancer: how are they linked? Free Radic Biol Med. 2010;49:1603–16. 10.1016/j.freeradbiomed.2010.09.006. 20840865 10.1016/j.freeradbiomed.2010.09.006 PMC2990475

[10] Schieber M, Chandel NS. ROS function in redox signaling and oxidative stress. Curr Biol. 2014;24:R453–62. 10.1016/j.cub.2014.03.034. 24845678 10.1016/j.cub.2014.03.034 PMC4055301

[11] Gorrini C, Harris IS, Mak TW. Modulation of oxidative stress as an anticancer strategy. Nat Rev Drug Discov. 2013;12:931–47. 10.1038/nrd4002. 24287781 10.1038/nrd4002

[12] Trachootham D, Alexandre J, Huang P. Targeting cancer cells by ROS-mediated mechanisms: a radical therapeutic approach? Nat Rev Drug Discov. 2009;8:579–91. 10.1038/nrd2803. 19478820 10.1038/nrd2803

[13] Glyn T, Williams S, Whitehead M, et al. Digital spatial profiling identifies molecular changes involved in development of colitis-associated colorectal cancer. Front Oncol. 2024;14:1247106. 10.3389/fonc.2024.1247106. 38505585 10.3389/fonc.2024.1247106 PMC10949367

[14] Levy JJ, Zavras JP, Veziroglu EM, et al. Identification of spatial proteomic signatures of colon tumor metastasis: a digital spatial profiling approach. Am J Pathol. 2023;193:778–95. 10.1016/j.ajpath.2023.02.020. 37037284 10.1016/j.ajpath.2023.02.020 PMC10284031

[15] Cancer Genome Atlas Network. Comprehensive molecular characterization of human colon and rectal cancer. Nature. 2012;487:330–7. 10.1038/nature11252. 22810696 10.1038/nature11252 PMC3401966

[16] Weinstein JN, Collisson EA, Mills GB, et al. The cancer genome atlas pan-cancer analysis project. Nat Genet. 2013;45:1113–20. 10.1038/ng.2764. 24071849 10.1038/ng.2764 PMC3919969

[17] Zhu J, Sanborn JZ, Benz S, et al. The UCSC cancer genomics browser. Nat Methods. 2009;6:239–40. 10.1038/nmeth0409-239. 19333237 10.1038/nmeth0409-239 PMC5027375

[18] Haam JH, Kim BT, Kim EM, et al. Diagnosis of obesity: 2022 update of clinical practice guidelines for obesity by the Korean Society for the Study of Obesity. J Obes Metab Syndr. 2023;32:121–9. 10.7570/jomes23031. 37386771 10.7570/jomes23031 PMC10327686

[19] Lee JG, Park I, Lee H, et al. Integrating E-cadherin expression levels with TNM staging for enhanced prognostic prediction in colorectal cancer patients. BMC Cancer. 2025;25:150. 10.1186/s12885-025-13539-9. 39871234 10.1186/s12885-025-13539-9 PMC11770905

[20] Kim S, Kang M, Jeong S, et al. Elucidating prognostic significance of purine metabolism in colorectal cancer through integrating data from transcriptomic, immunohistochemical, and single-cell RNA sequencing analysis. Mol Oncol. 2025. 10.1002/1878-0261.70010. 40017120 10.1002/1878-0261.70010 PMC12330927

[21] Wu G, Haw R. Functional interaction network construction and analysis for disease discovery. Methods Mol Biol. 2017;1558:235–53. 10.1007/978-1-4939-6783-4_11. 28150241 10.1007/978-1-4939-6783-4_11

[22] Chiolero A, Jacot-Sadowski I, Faeh D, et al. Association of cigarettes smoked daily with obesity in a general adult population. Obesity. 2007;15:1311–8. 10.1038/oby.2007.153. 17495208 10.1038/oby.2007.153

[23] Dare S, Mackay DF, Pell JP. Relationship between smoking and obesity: a cross-sectional study of 499,504 middle-aged adults in the UK general population. PLoS ONE. 2015;10:e0123579. 10.1371/journal.pone.0123579. 25886648 10.1371/journal.pone.0123579 PMC4401671

[24] Bardou M, Rouland A, Martel M, et al. Review article: obesity and colorectal cancer. Aliment Pharmacol Ther. 2022;56:407–18. 10.1111/apt.17045. 35707910 10.1111/apt.17045

[25] Brierley J, O’Sullivan B, Asamura H, et al. Global consultation on cancer staging: promoting consistent understanding and use. Nat Rev Clin Oncol. 2019;16:763–71. 10.1038/s41571-019-0253-x. 31388125 10.1038/s41571-019-0253-x PMC7136160

[26] Li S, Zhuang Z, Wu T, et al. Nicotinamide nucleotide transhydrogenase-mediated redox homeostasis promotes tumor growth and metastasis in gastric cancer. Redox Biol. 2018;18:246–55. 10.1016/j.redox.2018.07.017. 30059901 10.1016/j.redox.2018.07.017 PMC6079569

[27] Rydström J. Mitochondrial NADPH, transhydrogenase and disease. Biochim Biophys Acta. 2006;1757:721–6. 10.1016/j.bbabio.2006.03.010. 16730324 10.1016/j.bbabio.2006.03.010

[28] Kaludercic N, Di Lisa F. The energetic cost of NNT-dependent ROS removal. J Biol Chem. 2020;295:16217–8. 10.1074/jbc.H120.016368. 33246940 10.1074/jbc.H120.016368 PMC7705306

[29] Ward NP, Kang YP, Falzone A, et al. Nicotinamide nucleotide transhydrogenase regulates mitochondrial metabolism in NSCLC through maintenance of Fe-S protein function. J Exp Med. 2020. 10.1084/jem.20191689. 32196080 10.1084/jem.20191689 PMC7971138

[30] Sies H, Berndt C, Jones DP. Oxidative Stress. Annu Rev Biochem. 2017;86:715–48. 10.1146/annurev-biochem-061516-045037. 28441057 10.1146/annurev-biochem-061516-045037

[31] Sullivan LB, Chandel NS. Mitochondrial reactive oxygen species and cancer. Cancer Metab. 2014;2:17. 10.1186/2049-3002-2-17. 25671107 10.1186/2049-3002-2-17 PMC4323058

[32] Arnér ES, Holmgren A. The thioredoxin system in cancer. Semin Cancer Biol. 2006;16:420–6. 10.1016/j.semcancer.2006.10.009. 17092741 10.1016/j.semcancer.2006.10.009

[33] Ju H-Q, Lin J-F, Tian T, et al. NADPH homeostasis in cancer: functions, mechanisms and therapeutic implications. Signal Transduct Target Ther. 2020;5:231. 10.1038/s41392-020-00326-0. 33028807 10.1038/s41392-020-00326-0 PMC7542157

[34] Ciccarone F, Di Leo L, Lazzarino G, et al. Aconitase 2 inhibits the proliferation of MCF-7 cells promoting mitochondrial oxidative metabolism and ROS/FoxO1-mediated autophagic response. Br J Cancer. 2020;122:182–93. 10.1038/s41416-019-0641-0. 31819175 10.1038/s41416-019-0641-0 PMC7051954

[35] López-Grueso MJ, Lagal DJ, García-Jiménez ÁF, et al. Knockout of PRDX6 induces mitochondrial dysfunction and cell cycle arrest at G2/M in HepG2 hepatocarcinoma cells. Redox Biol. 2020;37:101737. 10.1016/j.redox.2020.101737. 33035814 10.1016/j.redox.2020.101737 PMC7554216

[36] Choi EH, Park SJ. TXNIP: a key protein in the cellular stress response pathway and a potential therapeutic target. Exp Mol Med. 2023;55:1348–56. 10.1038/s12276-023-01019-8. 37394581 10.1038/s12276-023-01019-8 PMC10393958

[37] Dong G, Wang Q, Wen M, et al. DDX18 drives tumor immune escape through transcription-activated STAT1 expression in pancreatic cancer. Oncogene. 2023;42:3000–14. 10.1038/s41388-023-02817-0. 37620449 10.1038/s41388-023-02817-0

