# Supplementary figures and images for: Multi-modal molecular and spatial profiling reveals NNT as a prognostic biomarker in obesity-associated colorectal cancer
Source: J Gastroenterol. 2025 Dec 28;61(4):435–49. doi: 10.1007/s00535-025-02339-4 (PMC13048935; doi:10.1007/s00535-025-02339-4)

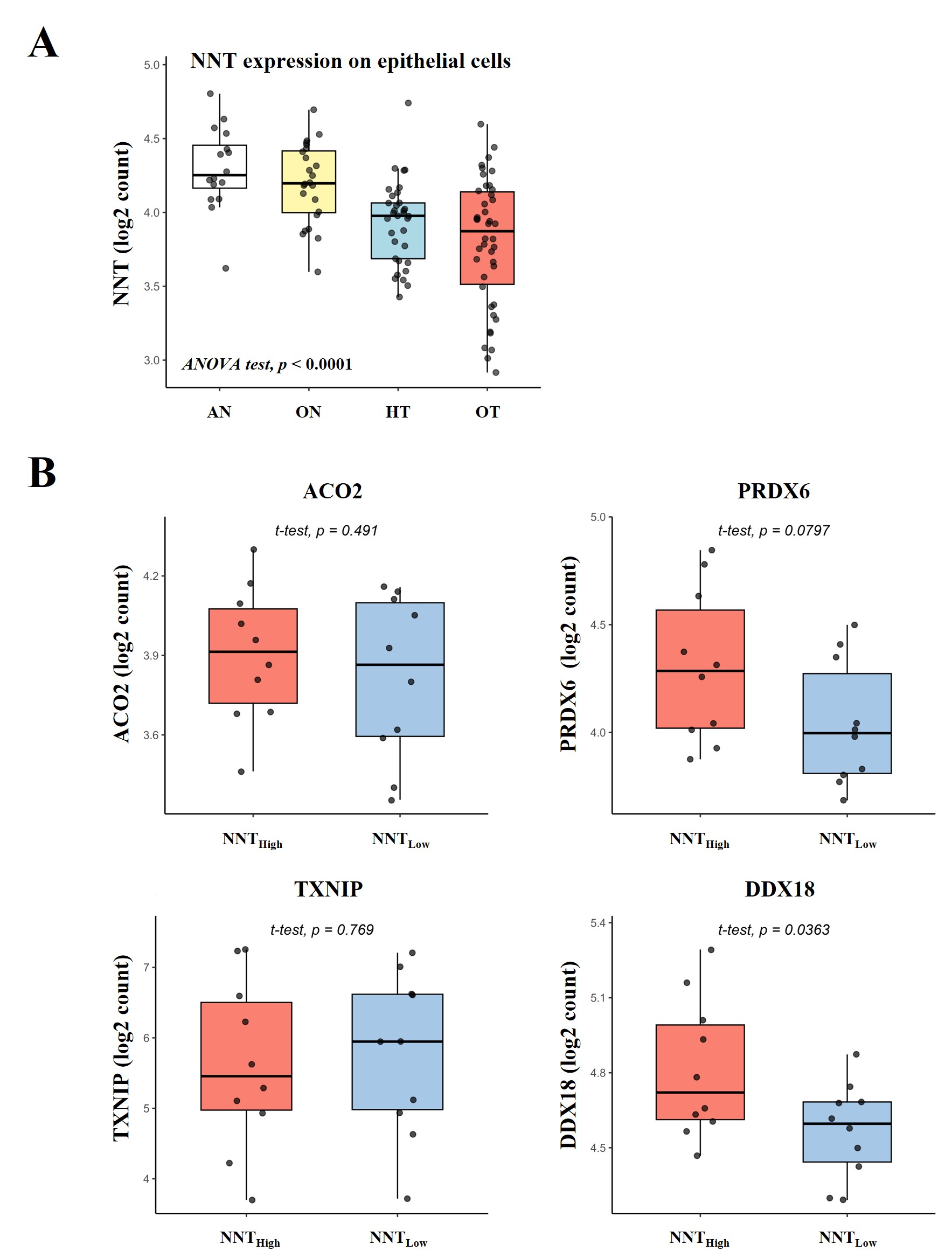

Supplement: Supplementary file 1 — Supplementary file1 Fig. S1 (A) NNT expression across AN, ON, HT, and OT (boxplot with ANOVA p-value, ROI numbers). (B) HT compartment gene expression (DDX18, PRDX6, TXNIP, ACO2) by NNT level (t-test p-values) (JPG 259 KB) [file 535_2025_2339_MOESM1_ESM.jpg]
